# Folate Receptor Alpha Expression in Lung Cancer: Diagnostic and Prognostic Significance

**DOI:** 10.18632/oncotarget.519

**Published:** 2012-04-29

**Authors:** Daniel J. O'Shannessy, Gordon Yu, Robert Smale, Yao-Shi Fu, Sunil Singhal, Robert P. Thiel, Elizabeth B. Somers, Anil Vachani

**Affiliations:** ^1^ Department of Diagnostics Development, Morphotek, Inc., Exton, PA; ^2^ Department of Medicine, University of Pennsylvania School of Medicine, Philadelphia, PA; ^3^ Department of Pathology, University of Pennsylvania School of Medicine, Philadelphia, PA; ^4^ Department of Surgery, University of Pennsylvania School of Medicine, Philadelphia, PA; ^5^ Laboratory Corporation of America, Los Angeles, CA; ^6^ Thiel Statistical Consulting, Oxford, CT

**Keywords:** folate receptor alpha, FRA, non-small cell lung cancer, adenocarcinoma, prognosis, immunohistochemistry

## Abstract

With the advent of targeted therapies directed towards folate receptor alpha, with several such agents in late stage clinical development, the sensitive and robust detection of folate receptor alpha in tissues is of importance relative to patient selection and perhaps prognosis and prediction of response. The goal of the present study was to evaluate the expression of folate receptor alpha in non-small cell lung cancer specimens to determine its frequency of expression and its potential for prognosis. The distribution of folate receptor alpha expression in normal tissues as well as its expression and relationship to non-small cell lung cancer subtypes was assessed by immunohistochemistry using tissue microarrays and fine needle aspirates and an optimized manual staining method using the recently developed monoclonal antibody 26B3. The association between folate receptor alpha expression and clinical outcome was also evaluated on a tissue microarray created from formalin fixed paraffin embedded specimens from patients with surgically resected lung adenocarcinoma. Folate receptor alpha expression was shown to have a high discriminatory capacity for lung adenocarcinomas versus squamous cell carcinomas. While 74% of adenocarcinomas were positive for folate receptor alpha expression, our results found that only 13% of squamous cell carcinomas were FRA positive (p<0.0001). In patients with adenocarcinoma that underwent surgical resection, increased folate receptor alpha expression was associated with improved overall survival (Hazard Ratio 0.39, 95% CI 0.18-0.85). These data demonstrate the diagnostic relevance of folate receptor alpha expression in non-small cell lung cancer as determined by immunohistochemistry and suggest that determination of folate receptor alpha expression provides prognostic information in patients with lung adenocarcinoma.

## INTRODUCTION

Folate receptor alpha (FRA), a glycosylphosphatidylinositol (GPI)-anchored cell surface glycoprotein, is a member of a family of folate receptors that mediate folate transport into cells [1]. The family consists of four isoforms (alpha, FRA; beta, FRB; gamma, FRG and delta, FRD). The FRA and FRB isoforms are highly homologous, GPI-anchored proteins that have high affinity (K_D_ ~1 nM) for 5-methyltetrahydrofolate, the predominant plasma folate [1]. These two family members, however, have distinct patterns of expression in normal and malignant tissues. Previous studies have found FRA to be mainly expressed on the apical surface of a limited subset of polarized epithelial cells in normal tissue and in certain malignancies of epithelial origin [2]. FRB expression has been reported to be restricted to hematopoietic cells of myelogenous lineage [3].

Due to its restricted expression pattern in normal and malignant cells, FRA is the most widely studied member of this folate receptor family [4]. Previous studies have found it to be highly expressed in non-mucinous (serous) ovarian and endometrial carcinomas, lung adenocarcinoma and to a lesser extent in breast cancer. Moreover, several studies have suggested that levels of FRA expression are associated with tumor stage and/or survival in some cancers, including ovarian cancer and lung adenocarcinoma [5-8].

The potential to exploit the differential expression of FRA for targeted cancer therapy has long been appreciated. Two primary approaches have been explored; one involving targeted drug delivery via folate-conjugated therapeutic compounds [9-12], and the other involving direct targeting and tumor cell death via humanized anti-FRA monoclonal antibodies [13-15]. Both approaches have advanced to late-stage clinical development, and anti-FRA monoclonal antibody therapy with farletuzumab is in clinical trials in both ovarian cancer and non-small cell lung adenocarcinoma.

Identification of patients who may benefit from FRA-targeted therapy may support the development of such therapeutics, particularly in cancers where the frequency and degree of expression is not ubiquitous or variable [16-18]. Detection of FRA on formalin-fixed paraffin embedded (FFPE) tissue sections via immunohistochemistry (IHC) requires highly sensitive and specific reagents. The development and characterization of novel, high-affinity FRA-specific antibodies that can be used in multiple diagnostic platforms, including IHC, has recently been described [19]. One unique clone, monoclonal antibody 26B3.F2 (MAb 26B3) displayed high-affinity FRA binding and the ability to recognize FRA in both its native and denatured forms [19].

Previous reports that FRA expression may vary by non-small cell lung cancer (NSCLC) histological subtype and that expression levels may be associated with disease stage or survival in lung adenocarcinoma suggest that FRA may be a useful diagnostic and prognostic marker [5, 6]. There is increasing evidence that histological subtype is an important determinant of treatment response in NSCLC. For patients with lung adenocarcinoma, multiple new treatment options have become available in the last few years, including agents such as pemetrexed and bevacizumab. Thus, standard-of-care practice now requires the performance of an expanded panel of IHC analyses in all cases of newly diagnosed NSCLC in order to distinguish between the major histologic subtypes; however this distinction can be challenging, particularly in small biopsy or cytological (e.g. fine needle aspiration or FNA) specimens.

Here we report the IHC characterization of MAb 26B3 in NSCLC and describe its ability to reliably discriminate between lung adenocarcinoma and squamous cell carcinoma. Furthermore, we describe the role of tumor FRA expression in the prognosis of patients with surgically resected lung adenocarcinoma.

## MATERIALS AND METHODS

### Tissue Microarrays

Normal tissue (catalog # FDA806-1; 3 individuals per tissue type) and lung carcinoma (catalog # BC041114; 90 cases, duplicate cores) TMAs were obtained from US Biomax, Inc. (Rockville, MD). These TMAs were used to define the distribution of FRA expression on a wide range of normal tissues and various histological types of lung cancer. To assess FRA expression as a prognostic indicator, an analysis was performed on a separate TMA constructed from tissues obtained from 68 subjects with newly diagnosed lung adenocarcinoma recruited from the University of Pennsylvania (Penn) from 2003 through 2006. The Penn TMA was constructed from FFPE archival tumor and adjacent normal specimens collected at the time of surgical resection. All tumors were reviewed by a lung pathologist for confirmation of histologic subtype. Three samples (0.6 mm cores) of representative tumor tissue and adjacent normal tissue from each subject and normal control tissues were prepared using a manual arrayer (Beecher Instruments, Inc., Sun Prairie, WI). A total of 15 subjects were excluded from the survival analysis because they had received treatment with neoadjuvant chemotherapy prior to surgery (6 subjects), lacked tumor cells in the TMA core (5 subjects) or lacked survival data (4 subjects). No other subjects received any therapy prior to surgical resection. Clinical outcome data was collected prospectively via clinic follow-up, review of electronic medical records, direct phone contact, and the Social Security Death Index (SSDI). Tissue samples from nine additional subjects with lung adenocarcinoma diagnosed by fine needle aspiration (FNA) of a subcarinal, hilar or paratracheal lymph node and containing sufficient material for preparation of paraffin-embedded cell blocks were used to determine the suitability of FNAs for the detection of FRA by IHC using MAb 26B3. The Penn Institutional Review Board approved this study and all subjects provided written informed consent for use of their tissues.

### Immunohistochemistry

IHC was performed using FFPE specimens and a MACH4 Universal HRP-Polymer Detection Kit (Biocare Medical, Concord, CA). FFPE specimens were sectioned at 5μm onto positively-charged glass slides and heated for approximately 60min at 60°C. Slides were deparaffinized in 3 sequential baths of xylene for 3min each, transferred to three sequential baths of 100% alcohol for 3min each, followed by three sequential baths of 95% alcohol for 3min each and then rinsed for 5min in deionized (DI) water. Slides were then pretreated in Diva heat-induced epitope retrieval solution (Biocare Medical) diluted 1:10 in DI water and placed inside a pressurized decloaking chamber already filled with 500mL of DI water. For antigen retrieval, slides were incubated for 15min inside the decloaking chamber in which pressurized incubation reaches a maximum of 125°C at 16 PSI for 30sec and then cooled for 15min down to 95°C. After cooling to room temperature (RT), slides were washed in 3 sequential baths of Tris Buffered Saline/0.1% Tween-20 wash buffer (TBST) for 3min each and subsequently placed into Peroxidase-1 (Biocare Medical) blocking solution for 5min at RT. After washing in TBST as above, Background Sniper (Biocare Medical) serum-free universal blocking reagent was applied for 10min at RT. Slides were then incubated with purified MAb 26B3.F2[19] or clone BN3.2[20] at 2.5μg/mL diluted in Antibody Diluent (Dako North America, Inc., Carpinteria, CA) or Universal Negative Control [mouse ready-to-use negative control antibody (Dako, for negative isotype tissue)] for 60min at RT. After washing, slides were incubated with MACH4 Mouse Probe Primary Antibody Enhancer for 15min, followed by Polymer-HRP reagent for 20min, developed with a 3,3′-diaminobenzidine tetrahydrochloride (DAB) solution (Dako) for 5min and counterstained with hematoxylin (Dako) for 2min, all incubations being performed at RT. Scoring for staining was performed by a single board-certified pathologist, using customary scoring for intensity and the percent of the tumor stained at each intensity.

### IHC Scoring method

**Figure 1:**
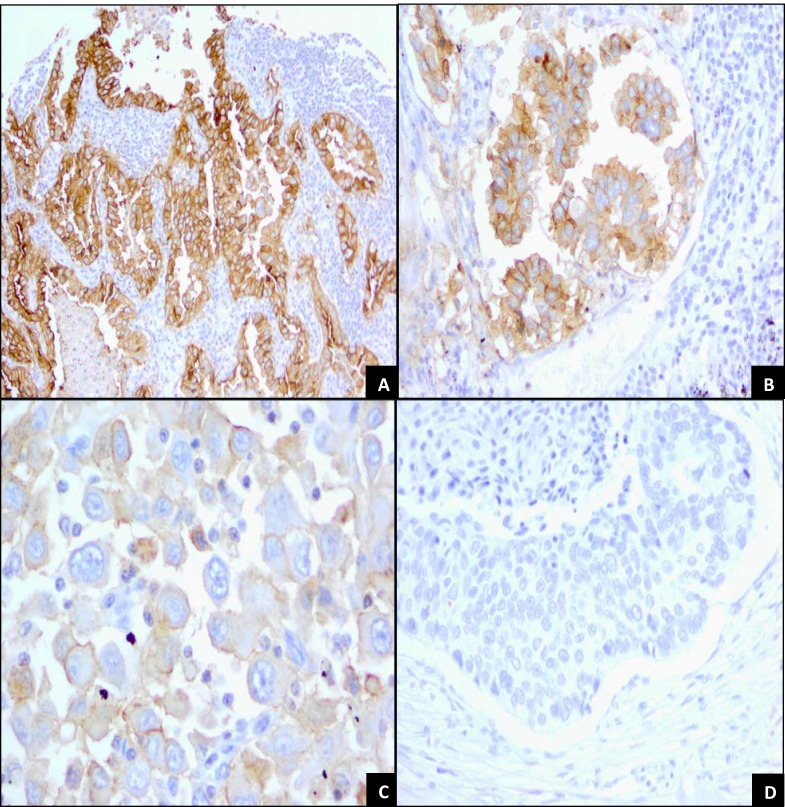
Staining Patterns and Intensities for FRA Expression in Lung Adenocarcinoma samples The figure illustrates and highlights the different staining patterns observed and the magnification required to accurately assess staining intensity. **A.** Lung adenocarcinoma with 3+, strong membrane staining on the luminal and lateral cell borders. Tumor cells in cross section reveal a honey-comb pattern (center field). Membrane staining is clearly visible by 10x objective. A weak cytoplasmic staining is present as well (10x). **B.** Lung adenocarcinoma. Clusters of tumor cells demonstrate 2+, moderate membrane staining on the luminal and lateral cell borders. Membrane staining is weaker and thinner than 3+ seen in Panel A (20x). **C.** Poorly differentiated adenocarcinoma. Tumor cells present with weak, incomplete membrane staining (40x). **D.** Lung squamous cell carcinoma. Solid sheet of tumor cells with necrotic, keratinized cells in the upper field. No membrane or cytoplasmic staining is seen (20x).

In this study, FRA IHC membrane and cytoplasmic staining intensities were scored as 0, no staining; 1+, weak; 2+, moderate and 3+, strong (Figure 1). The percent of cells in each core stained by MAb 26B3 (or an unrelated anti-FRA MAb called clone BN3.2) was also determined and recorded (see below). Samples were analyzed under 4x, 10x, 20x and 40x objectives. 3+ strong membrane staining was readily visualized under 4x and confirmed at 10x. 2+ moderate membrane staining was visible at 10x and confirmed at 20x and 1+ weak membrane staining required 20x or 40x objectives. In the presence of 3+ staining, the membrane was thick occurring at apical and lateral cell borders. In tangential sections, a complete circumferential pattern was evident (Figure 1A). 2+ membrane staining was weaker in intensity and thinner than 3+, usually localized on the apical luminal borders and occasionally on lateral cell borders (Figure 1B). 1+ weak membrane staining was generally limited to the luminal borders (Figure 1C). The accompanying cytoplasmic staining was variable, depending on the type of tumor, and although recorded, was not assessed further.

### Statistical Analyses

#### Criteria for a Positive Staining Result and TMA Core Rejection

A sample (TMA core or whole section) was considered positive for FRA expression if the percentage of the tumor area considered by the reading pathologist to be positive for membranous staining was greater than or equal to 5% at any intensity. A TMA core was rejected and therefore not included in the analyses if the reading pathologist determined it was either missing entirely (empty core), was composed of necrotic tissue or was deemed to represent normal tissue. Histopathologic diagnosis of cores was made by the reading pathologist.

#### The M-score: A Semi-Quantitative Staining Algorithm

A metric for staining (M-score) of each sample was defined as follows:

$$M_{i}=\frac{\sum_{j=1}^{3}W_{j}\cdot X_{ij}}{\sum_{j=1}^{3}W_{j}}=\frac{\sum_{j=1}^{3}W_{j}\cdot X_{ij}}{6}$$

In the equation, *x_ij_* is the percentage of tumor stained at intensity *j* for patient *i* and *w_j_* is the absolute value of the intensity (ranging from 0 to 3+). The metric has a theoretical range from zero (no positive staining) to fifty (100% of cells staining at 3+ intensity). As such, the M-score is a *weighted score* for FRA IHC tumor cell membrane staining that captures both the proportion of FRA positive cells and staining intensity.

M-scores for each patient were averaged over multiple TMA cores, where appropriate. If a determination (core) was void of results, *i.e.* no tumor present or necrotic tissue, the M-score was assigned to the non-void determinations.

The practical application of the above equation is presented below:

**Table:** 

3+	2+	1+	0	M-Score
x = 40	y = 30	z = 10	20	M = (3x+2y+z)/6
3 × 40 = 120	2 × 30 = 60	1 × 10 = 10	-	(120+60+10)/6=31.67

Here, × = % of tumor stained with intensity 3+; y = % of tumor stained with intensity 2+; z = % of tumor stained with intensity 1+.

The positivity rate for FRA expression within a given histology was calculated as the proportion of samples that were stained positive according to the definition of a positive result (≥5% of the total tumor cells staining). Exact binomial confidence intervals were determined using the methods of Clopper and Pearson.[21]

Summary statistics are presented for all demographic variables and for the M-score. Differences for mean values were determined using one-way ANOVA with *post-hoc* tests controlling for overall type I error. Differences in mean values were statistically different if the p-value associated with the test was less than the Bonferroni adjusted type I error for that test (maximum Type I error=0.05).

All metric analyses were performed using SPSS version 18 for Windows (IBM Corporation, Armonk, NY, 2009). Binomial confidence intervals were constructed using Excel (Microsoft Office, 2010, Microsoft Corporation, 2009)

#### Survival Analysis

For the subset of subjects in the survival analysis using the Penn TMA, an optimal cut-point for the M-score was determined by a receiver operating characteristic (ROC) analysis. Using the diagnostic likelihood ratio method as described by Pepe [22], we found that at an M-score cut-point of 10, the odds ratio (OR) for death achieved a maximum of 6.6. This value of the M-score was chosen to assign individuals to either a high FRA or low FRA expression category. The method of Kaplan-Meier was used to estimate overall survival curves based on high or low FRA expression. Multivariable cox proportional hazard models were used to adjust for potential confounding in the association between FRA level and overall survival. All survival analyses were performed in Stata 12 (StataCorp., College Station, TX).

## RESULTS

### Comparison of MAb 26B3 and Clone BN3.2 for Staining of Lung Carcinomas

**Figure 2:**
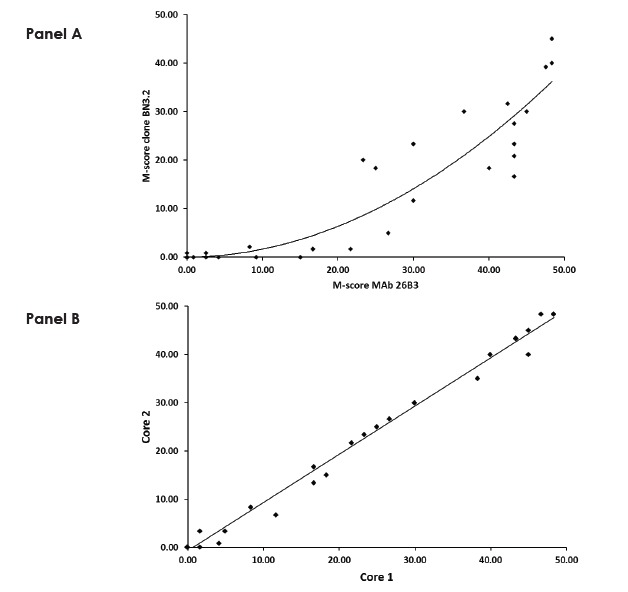
Comparison of M-scores for MAb 26B3 and clone BN3.2 for Lung Adenocarcinoma **A.** Scatterplot (MAb 26B3 *versus* clone BN3.2) demonstrating the increased sensitivity of MAb 26B3 for the detection of FRA expression on FFPE samples. The relationship is clearly non-linear and is best described by the equation: y = 0.0152x^2^ + 0.0151x − 0.0181; R^2^ = 0.8721 **B.** Scatterplot of M-scores determined using MAb 26B3 on duplicate cores of lung adenocarcinoma. The linear relationship is described by: y = 0.9999x - 0.6452; R^2^ = 0.9926

There is significant variation in the literature with respect to the percent of various carcinomas that express FRA as determined by IHC, in part due to the use of a variety of antibodies, most of which are not commercially available. One FRA specific MAb that is commercially available and has been demonstrated to detect FRA on FFPE sections by IHC is clone BN3.2 (Leica Microsystems, Buffalo Grove, IL) [20]. To assess the reproducibility between two distnct anti-FRA MAbs, we conducted IHC using clone BN3.2 and the recently developed MAb 26B3 for both specificity and sensitivity for the detection of FRA using the commercial TMA containing various histological types of lung cancer. Both antibodies were highly specific for adenocarcinoma as compared with other histologic subtypes, particularly squamous cell carcinoma (Figure 1). However, MAb 26B3 was significantly more sensitive than clone BN3.2, identifying 26/36 (72%; M-score mean ± SD = 19.84 ± 18.64) and 22/36 (61%; M-score mean ± SD = 11.38 ± 14.25) adenocarcinoma samples, respectively (p<0.0001). These data suggest that under the conditions employed here that clone BN3.2 is significantly less sensitive than MAb 26B3 for detecting FRA expression on FFPE tissues and, as shown in Figure 2A, the relationship in observed M-scores on lung adenocarcinoma samples for these two MAbs is clearly non-linear. All subsequent analyses described in the present work were performed using MAb 26B3, given its robust sensitivity in IHC under conditions described here.

### Normal Tissue Staining Distribution for MAb 26B3

**Figure 3:**
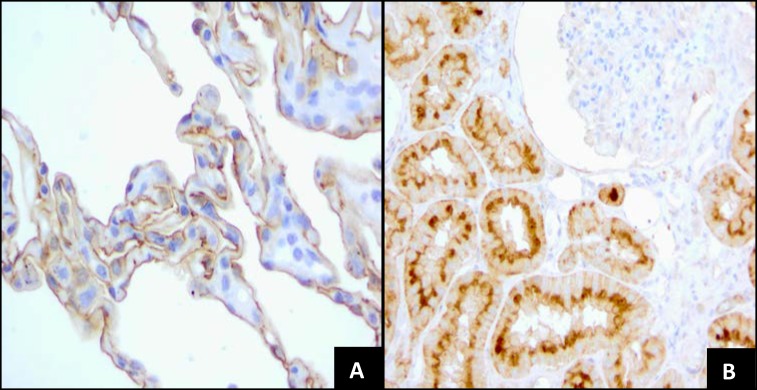
FRA Expression in Normal Tissues **A.** Normal lung tissue with 2+, moderate membrane staining on the surface of alveolar cells (40x). **B.** Normal kidney with 3+ strong, thick, membrane staining on the luminal borders of proximal convoluted tubular cells. Glomerular cells are negative (40x).

The pattern of FRA expression with MAb 26B3 on a normal tissue TMA was consistent with previously published literature using a variety of other antibodies and techniques [20, 23]. Pancreas, thyroid, lung, salivary gland, kidney, hypophysis, cervix and breast showed variable expression (Table 1). In Figure 3, the staining pattern in normal tissues, exemplified in normal kidney and normal lung sections, is highly restricted to epithelial cells and typically apical in nature. Such a staining pattern is consistent with the proposed biological functions of FRA in normal tissue. For example, FRA distributed on the proximal tubules of the kidney is thought to scavenge filtered folates and recycle them into the circulation so they are not lost into the urine [24, 25]. Similarly, FRA expression on bronchiolar epithelium is thought to have anti-bacterial activity by sequestering folates away from colonizing bacteria [26-28].

**Table 1:** FRA Expression (MAb 26B3) in Normal Human Tissues

Tissue Type	FRA Staining* (Number FRA +/Total; Intensity)	Comments
Cerebrum	0/3	
Cerebellum	0/3	
Adrenal	0/3	
Ovary	0/3	
Pancreas	3/3; 2+	Limited to luminal borders of ductal and acinar cells
Thyroid	2/5; 1+ (sparse)	Cytoplasmic staining in follicular cells
Hypophysis	3/3; 1+	Predominantly cytoplasmic
Testis	0/3	
Breast	3/3; 1+/2+	Ductal cells with luminal and membrane staining
Spleen	0/3	
Tonsil	0/3	
Thymus	0/3	
Bone Marrow	0/3	
Lung	3/3; 2+	Staining in bronchial and alveolar cells
Heart	0/3	
Esophagus	0/3	
Stomach	0/2	
Small Intestine	0/3	
Colon	0/3	
Liver	0/3	
Salivary Gland	3/3; 3+	Ductal and acinar cells
Kidney	3/3; 3+	Luminal staining of proximal tubular cells
Prostate	0/3	
Endometrium	0/3	
Cervix	1/3; 1+	Endocervical cells
Skeletal Muscle	0/3	
Skin	0/3	
Nerve	0/3	
Mesothelium	0/3	

* The table shows FRA staining results for multiple normal human tissue specimens and is displayed as the number of FRA + specimens/total number of specimens analyzed for each tissue type. Each specimen represents a unique individual. Intensity, noted as 3+ (strong), 2+ (moderate) or 1+ (weak) is an *average* staining intensity across all positive samples as determined by the reading pathologist.

### FRA Discriminates Between Lung Adenocarcinomas and Lung Squamous Cell Carcinomas

**Figure 4:**
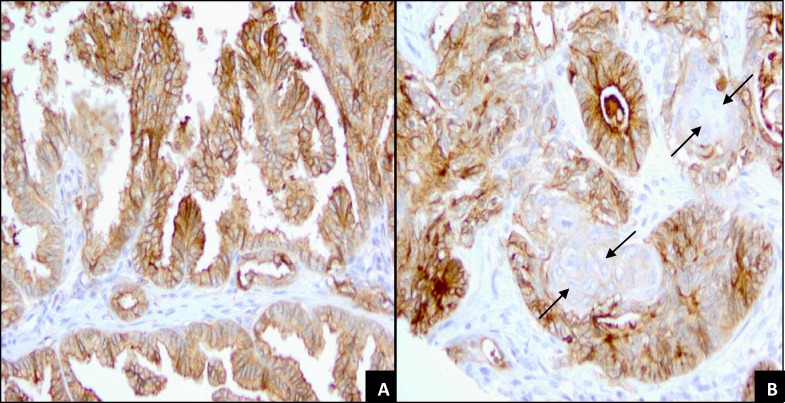
FRA Expression of papillary adenocarcinoma and adenosquamous carcinoma **A.** Lung papillary adenocarcinoma with 70% of tumor cells showing 3+ membrane staining and 30% 2+ membrane staining (20x). **B.** Lung adenosquamous carcinoma. Whorls of squamous cells (arrows) are surrounded by tall columnar cells with 3+ membrane staining. Malignant squamous cells are entirely negative for membranous staining, although there is a weak cytoplasmic staining. (20x).

We evaluated a total of 89 lung cancer samples on the commercial TMA: 36 (40%) were adenocarcinoma, 32 (36%) were squamous cell carcinoma, 2 (2%) were adenosquamous carcinomas, and the remaining 19 (21%) were of a variety of other histologies (Table 2). The overall rates of FRA positivity varied substantially for each of the histologic subtypes. A significantly higher proportion of adenocarcinoma tumors were positive for FRA when compared to squamous cell carcinomas (72% *versus* 13%, p<0.0001). Of the 4 positive squamous cell carcinoma samples, only 1 showed 3+ staining on both cores; 1 had intermediate (2+) staining on both cores and the other 2 were very weakly positive in a single core (5-10% of tumor cells at 1+). The two adenosquamous carcinoma samples present on this TMA were also shown to be positive for FRA, with staining (2+ - 3+) restricted to the adenocarcinoma portion of these samples (Figure 4).

**Table 2:** Distribution of FRA Expression^1^

Variable	FRA negative N (%)	FRA positive N (%)	Total	P value^2^
				
**Tumor Histology**				
Normal	1 (10%)	9 (90%)	10	
Squamous cell carcinoma	28 (87%)	4 (14%)	32	<0.0001
Large cell carcinoma	3 (60%)	2 (40%)	5	
Small cell carcinoma	7 (87%)	1 (13%)	8	
Neuroendocrine carcinoma	4 (67%)	2 (33%)	6	
Adenocarcinoma^3^	10 (16%)	28 (74%)	38	
**Tumor Grade**				
Grade 1	1 (20%)	4 (80%)	5	
Grade 2	5 (22%)	18 (78%)	23	
Grade 3	4 (40%)	6 (60%)	10	0.517
**Tumor Stage**				
Stage I	4 (29%)	11 (71%)	15	
Stage II	2 (17%)	10 (83%)	12	
Stage III + IV^4^	4 (36)	7 (64)	11	0.563
**Gender**				
Female	3 (18%)	14 (82%)	17	
Male	7 (33%)	14 (67%)	21	0.46

1 US Biomax Lung Cancer TMA

2 p-values determined using Fisher’s exact test or chi-square test: squamous cell carcinoma *versus* adenocarcinoma p<0.0001; males *versus* female, p=0.46; stage, p=0.563; grade, p=0.517

3 Includes 2 adenosquamous cases, both positive for FRA in the adenocarcinoma portion only

4 Only 1 stage IV case

The staining of duplicate cores in the adenocarcinoma samples was very consistent, (Figure 2B), a reflection of the robustness of MAb 26B3 staining. An examination of M-scores by stage and grade within the adenocarcinoma histologic subtype indicated that neither stage nor grade of disease was associated with the degree of FRA expression (data not shown).

**Figure 5:**
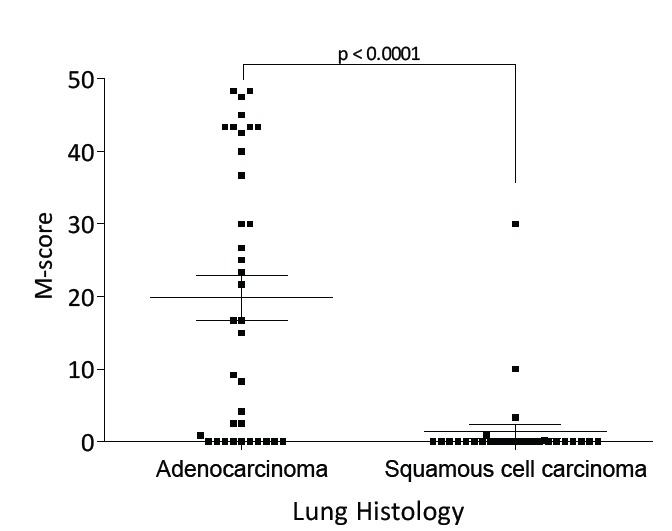
M-score Distribution of FRA Expression in Lung Adenocarcinoma and Squamous Cell Carcinoma M-scores by histology type for FRA expression by IHC using MAb 26B3 were calculated as described. The mean (± SEM) M–scores were 19.84 (± 18.64) for adenocarcinoma and 1.39 (± 5.54) for squamous cell carcinoma, respectively (p<0.0001).

The M-score distribution for FRA staining of lung adenocarcinoma and squamous cell carcinoma samples is shown in Figure 5. The mean (± SEM) M-scores for adenocarcinoma and squamous cell carcinoma samples stained with MAb 26B3 were 19.84 (± 18.64) and 1.39 (± 5.54), respectively (p<0.0001). The M-score for adenocarcinoma samples was also significantly higher when compared against all other lung cancer histologic types. In addition, we performed a Tree Analysis to determine the odds for the histology of the cancer being adenocarcinoma. An M-score >21.7 resulted in an odds ratio (OR) of 16, further demonstrating that FRA is predominately expressed in the adenocarcinoma histology (analysis not shown).

### Fine Needle Aspiration Provides Sufficient Material for FRA Staining

**Figure 6:**
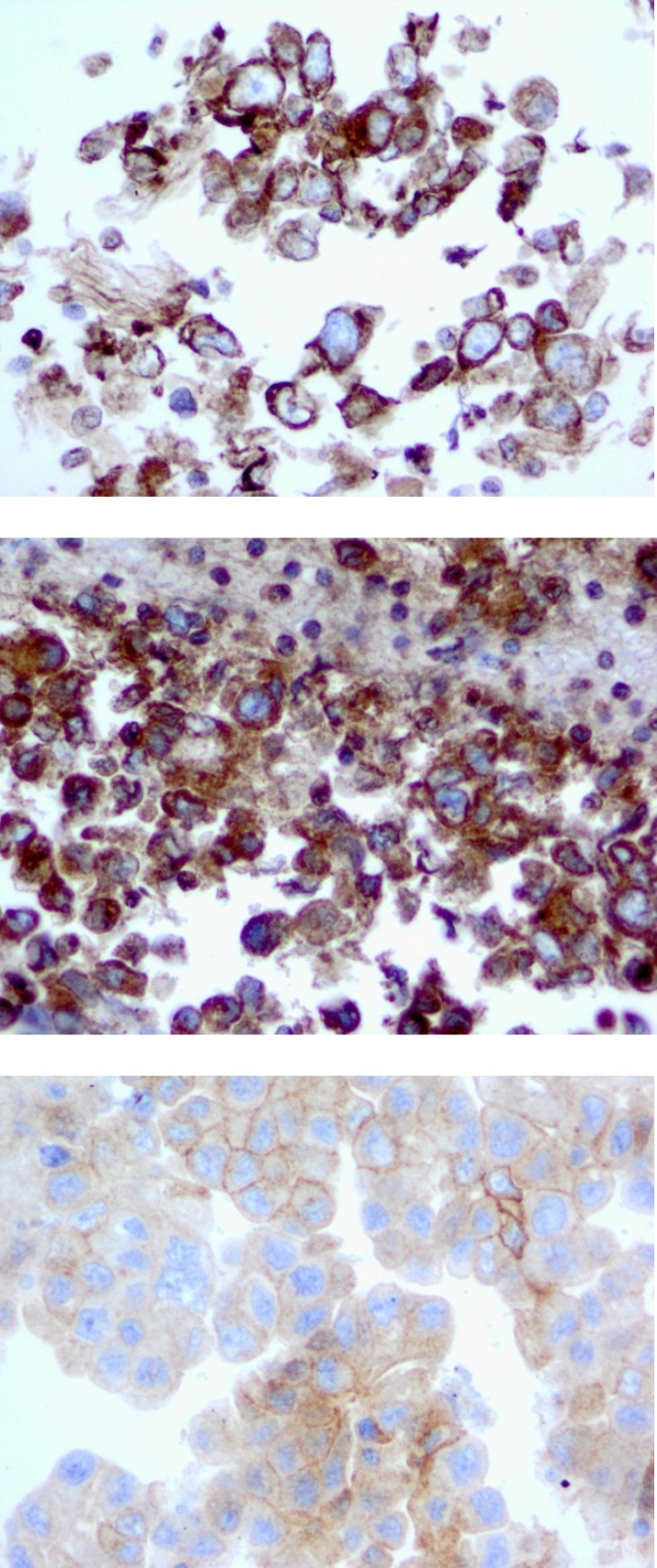
FRA Expression in Lung Adenocarcinoma FNA samples Staining of cell block material from lymph node FNAs with MAb 26B3 demonstrates successful staining of FRA, with expression limited to epithelial cells in an apical distribution.

FFPE tissue blocks are rarely available from patients diagnosed with late stage lung cancer, as surgical resections are not typically performed; however biopsies in these patients are usually performed via small core specimens or fine needle aspirates (FNA). Therefore, we assessed the suitability of FRA IHC using MAb 26B3 on cytology material obtained by FNA. We used samples from nine late-stage adenocarcinoma patients diagnosed by cytological evaluation of a thoracic lymph node aspirate (Figure 6) and demonstrated that the rate of FRA positivity (63%) was comparable to that seen for the histological specimens assessed on the lung cancer TMA (Table 2). Although only a small sample size, these data suggest that cytologic specimens may be a suitable tissue source for determining FRA expression in late stage adenocarcinoma patients.

### FRA Positivity and Survival in Lung Adenocarcinoma

**Figure 7:**
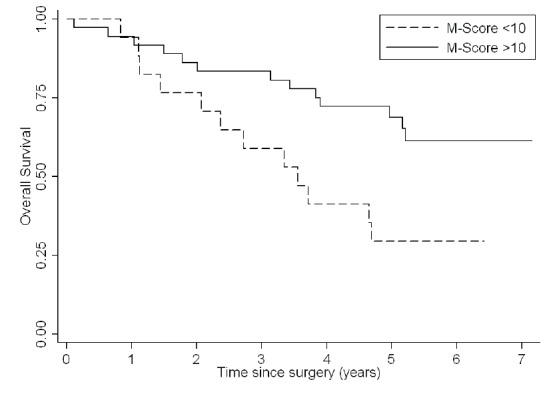
Overall Survival Curves for Lung Adenocarcinoma Patients by M-score Patients with high FRA expression (M-score ≥ 10) survived for a longer period when compared with low FRA-expression (M-score < 10).

Clinical characteristics and FRA expression results for the 55 subjects with newly diagnosed lung adenocarcinoma on the Penn TMA and included in the outcome analysis are listed in Table 3. The reading pathologist was blinded to the clinical information when analyzing the FRA staining. All subjects were treated with surgical resection. Of the 55 subjects, 30 (57%) were alive at five years. To determine if FRA expression was associated with overall survival, we first used ROC analysis to determine an optimal cut-off for the M-score, which identified an M-score of 10 as the FRA level that provided the best discrimination in overall survival. High FRA expression (M-score ≥10) was associated with improved overall survival (HR 0.39, 95% CI 0.18-0.85; Figure 7). Median survival for the low FRA expressers (M-score < 10) was 43.2 months. For the patients with high FRA expression (M-score ≥ 10), median survival was indeterminate because more than 50% of the subjects were still alive at the end of follow-up. Higher FRA expression remained significantly associated with overall survival after adjusting for stage, age, gender, and race (Table 4).

**Table 3:** Characteristics of Patients with Lung Adenocarcinoma on Penn TMA

Variable	Cases
**Number**	53
**Mean age, years (SD)**	63.4 (9.7)
**Sex, n (%)** **Male** **Female**	32 (60) 21 (40)
**Race, n (%)** **Caucasian** **African-American** **Asian**	41 (77) 11 (21) 1 (2)
**Stage, n (%)** **IA** **IB** **IIA** **IIB** **IIIA** **IIIB**	17 (32) 15 (28) 4 (8) 3 (6) 9 (17) 4 (8)
**FRA Expression, n (%)** **M-Score ≥ 10** **M-Score < 10**	36 (68) 17 (32)

**Table 4:** Multivariable Cox Regression Models Demonstrating Adjusted HR for Overall Survival

Variable	HR (95% CI)	P value
**FRA expression (base model)^1^**	0.39 (0.18-0.75)	0.018
**Adjusted for:**		
**Stage**	0.39 (0.18-0.75)	0.018
**Age**	0.42 (0.19-0.94)	0.034
**Gender**	0.39 (0.18-0.86)	0.019
**Race**	0.40 (0.17-0.91)	0.029

A HR of less than 1 indicates that the factor is associated with better overall survival. Potential confounders were added one covariate at a time.

1 Hazard ratio for FRA M-score ≥10 compared to M-score <10

## DISCUSSION

Treatments for patients with non-small cell lung cancer have undergone a paradigm shift in the last 5 years. Previously, the most critical clinical distinction was to discriminate between small cell carcinoma and NSCLC as there was no clear therapeutic or prognostic reason to differentiate between the heterogeneous tumors comprising NSCLC (predominately adenocarcinoma and squamous cell carcinoma). The emergence of targeted therapies for specific histologic subtypes of lung cancer has led to an improvement in survival rates in both recurrent and metastatic NSCLC. It is increasingly apparent that both pathologic analysis and molecular subtyping will be necessary in determining the appropriate therapy for individual patients. For example, bevacizumab, a monoclonal antibody targeting vascular endothelial growth factor, in combination with standard chemotherapy, improves survival in non-squamous histology, but is contraindicated in patients with squamous cell carcinoma [29, 30]. In addition, it has recently been shown that specific sub-histology is also important in determining response to chemotherapeutic agents such as gemcitabine (improved survival in squamous cell) and pemetrexed (improved survival in adenocarcinoma and large cell) [31].

The distinction between the various histologic subtypes in NSCLC remains an ongoing clinical challenge, particularly when only limited, cytology-based samples are available. Previous studies have demonstrated the precise distinction of various subtypes is particularly challenging in cytologic specimens such as fine needle aspirates of lymph nodes or primary lung lesions, due to limited tumor cellularity and the frequent absence of tissue architecture [32-34]. Most cytopathology laboratories currently employ an IHC panel in these cases as an adjunct to aid tumor sub-classification, including TTF-1, Napsin A (positive in adenocarcinoma), cytokeratin 5/6 and P63 (positive in squamous cell carcinoma). Given the high level expression of FRA in adenocarcinoma, in contrast to its very limited expression in squamous cell carcinoma, the present data supports further studies on FRA expression and its potential inclusion in IHC panels used for sub-classification of NSCLC.

Most patients with early stage (I and II), and some with locally advanced disease (stage III), are treated by surgical resection with or without adjuvant chemotherapy. Although stage is the major determinant of prognosis, other factors are important in distinguishing the biology of these tumors. There has been a great deal of work in identifying molecular prognostic factors, with studies evaluating the role of factors such as gene copy number, mRNA expression, and most commonly, the role of protein expression using IHC [35, 36]. Previous studies in lung cancer with IHC markers have been largely inconsistent [36]. Thus, there is still a need to identify reliable and robust prognostic markers in this disease.

Given that the expression of FRA is largely limited to adenocarcinoma, we chose to evaluate the prognostic value of this marker on this patient population. In our analysis, we found that higher FRA expression was associated with a more favorable prognosis following surgical resection for lung adenocarcinoma. This finding is consistent with a previous study of surgically resected lung adenocarcinoma in which FRA expression was assessed at the mRNA level using quantitative PCR [7].

The mechanisms of increased FRA expression in cancer and its potential effect on prognosis are not well understood. While folic acid/vitamin B9 is essential to all mammalian cells, given its critical role in 1-carbon metabolism and methylation of nucleotides, lipids and proteins, FRA is apparently not required since its expression in normal tissues is highly restricted and its function more distinct than the ubiquitously expressed reduced folate receptor (RFC) (19). FRA has been clearly demonstrated to be essential during embryonic development but its role in some terminally differentiated adult tissues is, at best, speculative. On the other hand, RFC is responsible for the sequestration of folate into cells and for folate homeostasis. The expression of FRA over and above the expression of RFC has been postulated to impart a growth advantage to cells expressing this receptor, in part because of the very high affinity of FRA for folates relative to the RFC which may be advantageous in low folate environments or in situations of accelerated metabolic rate or growth, as in malignant tissue. It is interesting to speculate, therefore, that FRA expression on tumor cells imparts a growth advantage to those cells, especially since they are rapidly dividing and thus have increased folate requirements. Other functions for FRA include folate independent mechanisms whereby the receptor is thought to participate in transformation and increased cell growth via signal transduction with Lyn kinase members (13).

The question remains however, as to whether the expression of FRA is causative or simply a bystander in the pathogenesis of cancer. The expression of FRA on adenocarcinoma of the lung correlates well with the expression of FRA on normal type I and II pneumocytes in that adenocarcinomas frequently arise from these cell types and thus supports the hypothesis that a FRA expressing tumor is a result of the cell of origin of that tumor *naturally* expressing this receptor. This is further supported by the lack of expression of FRA in squamous cell carcinoma which derives from more centrally located respiratory epithelium that are universally negative for FRA expression in normal tissue. The small number of FRA expressing squamous cell carcinomas identified in the present study may represent a unique molecular subtype of NSCLC. The fact that not all adenocarcinomas appear to express FRA may be a result of assay sensitivity or, alternatively, FRA negative adenocarcinomas may represent a different epithelial cell of origin. Either way, FRA expression appears to identify a specific molecular subtype of lung adenocarcinoma. Additional studies may further elucidate not only the biology of FRA expression in lung adenocarcinoma but the prognostic significance of this receptor. Clinical trials currently underway in lung adenocarcinoma known to express FRA may help clarify this emerging area of investigation.
